# Concomitant Use of Analgesics and EGFR TKIs in Lung Cancer Patients: Outcomes and Perspectives From a Finnish Retrospective Register–Based Study

**DOI:** 10.1002/cam4.71040

**Published:** 2025-07-12

**Authors:** Laura S. Puuniemi, Sanna M. E. Iivanainen, Martti Arffman, Riitta L. Kaarteenaho, Jussi P. Koivunen

**Affiliations:** ^1^ Research Unit of Cancer and Translational Medicine, Cancer Center, Medical Research Center (MRC) Oulu University of Oulu and Oulu University Hospital Oulu Finland; ^2^ Department of Public Health and Welfare Finnish Institute for Health and Welfare Helsinki Finland; ^3^ Research Unit of Biomedicine and Internal Medicine, Center of Internal Medicine and Respiratory Medicine, Medical Research Center (MRC) Oulu Oulu University Hospital, University of Oulu Oulu Finland

**Keywords:** EGFR TKI, NSAID, NSCLC, opioid

## Abstract

**Introduction:**

Epidermal growth factor receptor (EGFR) tyrosine kinase inhibitors (TKIs) are used in the treatment of non‐small cell lung cancer (NSCLC). Preclinical studies suggest inflammatory and other mechanisms of analgesics affect the efficacy of EGFR TKIs. In this study, we aim to explore the outcomes of concurrent use of EGFR TKIs and analgesics, to provide clinical insight into analgesic treatment decisions.

**Methods:**

Patients (*n* = 1494) with EGFR TKI reimbursements (2011–2020) and data available in the Finnish Cancer Registry with concurrent analgesics purchases (nonsteroidal anti‐inflammatory drugs [NSAID], acetaminophen, weak and strong opioids, strong opioids stratified by immunomodulatory properties) were identified. Overall survival (OS) and time‐on‐treatment (ToT) were analyzed using univariate and multivariate Cox models and Kaplan–Meier.

**Results:**

In multivariate analysis for ToT, weak and strong opioids were associated with inferior outcomes (HR 1.368, 95% CI 1.119–1.674, HR 1.454, 95% CI 1.276–1.656) compared to no analgesics, while NSAID and acetaminophen showed no association. Multivariate analysis for OS showed inferior survival among EGFR TKI‐treated patients with weak (HR 1.290, 95% CI 1.043–1.595) and strong opioid (HR 1.690, 95% CI 1.471–1.940) purchases, while this was not seen with NSAID. Compared to nonimmunomodulatory opioids, patients with immunomodulatory opioid purchases had unfavorable outcomes for both ToT (HR 1.448, 95% CI 1.148–1.826) and OS (HR 1.479, 95% CI 1.158–1.888).

**Conclusions:**

In EGFR TKI‐treated NSCLC, opioids are an independent risk factor for worse ToT and OS. The outcomes differed by immunomodulatory category of opioids, suggesting analgesics class can potentially have an impact on EGFR TKI effects.

## Graphical abstract

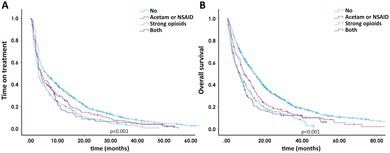

## 1 Introduction

Epidermal growth factor receptor (EGFR) tyrosine kinase inhibitors (TKI) are widely used in the treatment of advanced non‐small cell lung cancer (NSCLC). Initially, the TKI treatment was broadly applied in NSCLC, but in recent years, the use has been limited mostly to EGFR mutant lung cancers [1]. While EGFR TKIs have been extensively investigated in randomized studies and compared to standard‐of‐care chemotherapy, there is limited knowledge regarding the simultaneous use of other supportive medications and their impact on outcomes. Lung cancer patients often suffer from comorbidities and disease‐related symptoms and are affected by polypharmacy, which can influence treatment outcomes [2, 3, 4, 5, 6].

Most cancer patients suffer from pain during their treatments, and the use of analgesics is very common [7]. Furthermore, pain is associated with inferior cancer prognosis in advanced stage [8, 9, 10]. Many malignancies overexpress cyclooxygenase‐2 (COX‐2), and higher COX‐2 levels in tumors are associated with worse cancer prognosis in NSCLC [11, 12]. The use of nonsteroidal anti‐inflammatory drugs (NSAIDs), inhibitors of COX enzymes, has been studied for providing possible survival benefits. In a recent publication, superior survival was found in all NSCLC patients receiving NSAIDs [13]. A similar result was reported in an NSCLC population treated with immune checkpoint inhibitors (ICIs), yet to the best of our knowledge, no such studies on patients with EGFR TKIs exist [14].

Opioid usage improves performance status (PS) more compared to nonopioid analgesics, yet there is no evidence of opioids improving overall survival (OS), even though PS is shown to correlate with cancer survival [8, 15, 16]. On the contrary, prolonged opioid usage is independently associated with worse survival in cancer patients [8, 17, 18, 19, 20]. It is hypothesized that opioids, especially morphine, due to their immunomodulatory nature, might promote tumorigenesis through several mechanisms [20, 21, 22]. The immunomodulatory class of each opioid varies, and to our knowledge, no earlier work has reported differences in treatment outcomes between immunomodulatory and nonimmunomodulatory opioids [20, 21, 22, 23]. Furthermore, among EGFR TKI‐treated lung cancers, there is little clinical data available on the use of weak (tramadol, codeine, buprenorphine) or strong (morphine, oxycodone, fentanyl, hydromorphone) opioids and their effect on treatment outcomes.

In the current study, we aimed to investigate the outcomes of lung cancer patients concomitantly using EGFR TKIs and analgesics in a real‐world setting. We hypothesize that analgesic use is associated with time on treatment and survival in EGFR TKI‐treated lung cancer patients.

## 2 Methods

### 2.1 Patient Cohort

All the patients who received special reimbursement for EGFR TKIs (gefitinib, erlotinib, afatinib, and/or osimertinib) during the years 2011–2020 were collected from the Special Reimbursement Register of the Social Insurance Institution (SII) of Finland. During the study period, TKIs were reimbursed according to the following criteria: (1) gefitinib: presence of activating EGFR mutations; (2) afatinib: presence of activating EGFR mutations or 2nd or later treatment in squamous cell carcinoma; (3) erlotinib: 2nd or later line treatment or presence of activating EGFR mutations; (4) osimertinib: the presence of EGFR T790M. Using personal identity codes, data for the patients were linked from the Prescription database of SII, the Finnish Cancer Registry (FCR), and the Causes of Death Statistics of Statistics Finland from 2011 until the end of June 2021. Pseudonymization was performed by the Finnish Social and Health Data Permit Authority (Findata) before the data analysis. First‐line osimertinib patients (*n* = 4) were excluded from the study due to the small number of patients. The final analysis was carried out on patients (*n* = 1494) who had erlotinib, gefitinib, or afatinib reimbursement; had purchased EGFR TKIs; and had data available in FCR.

FCR does not include data on EGFR mutations. An artificial EGFR mutant cohort (*n* = 466) was generated according to reimbursement criteria, consisting of all gefitinib–treated patients and a subgroup of afatinib–treated patients without squamous cell histology.

Data on EGFR TKI and analgesic purchases were collected from the Prescription database of SII using Anatomical Therapeutic Chemical (ATC)‐codes. The first grouping was formed as follows: NSAID (including all COX‐2‐selective and nonselective NSAIDs in the indication of pain, topical products excluded), acetaminophen, weak opioids (codeine, buprenorphine, tramadol), and strong opioids (morphine, fentanyl, oxycodone, hydromorphone). Additionally, strong opioids were further divided into immunomodulatory (morphine, fentanyl) and nonimmunomodulatory (oxycodone, hydromorphone) opioids by their ability to affect immunological parameters [21, 22, 23]. Since the concomitant use of NSAID/acetaminophen and strong opioids is common, we also investigated the effect of concurrent use of both nonopioid and strong opioid analgesics. We divided our patient cohort into four groups: no analgesics, nonopioids (NSAID and acetaminophen purchases excluding those with concurrent opioids), strong opioids (concurrent nonopioids excluded), and users of both (including those with concomitant nonopioid and strong opioid purchases).

### 2.2 Outcomes

OS was analyzed from the 1st EGFR TKI purchase date to death or end‐of follow‐up, death counted as an event. Time‐on‐treatment (ToT) was analyzed from the date of the 1st EGFR TKI purchase to the last purchase date plus days on the treatment, according to the number of tablets in the last purchase. Treatment discontinuation before June 30, 2021 was counted as an event. However, a gap of 10 days between purchases was allowed to account for a continuation of the treatment.

Analgesic purchases were analyzed in a timeframe where 0 day was the 1st EGFR TKI purchase date. The time window −14 days to +14 days was selected to represent the initiation period of an EGFR treatment, and −14 days to +180 days was used to reflect a longer follow‐up. Because of the increasing length time bias with longer timeframes, multivariate analyzes were performed on the −14 days to +14 days groups only where applicable to ensure minimum disturbance and maximum convergence of the results.

### 2.3 Statistics

Since time‐dependent effect measures (TOT and OS) were used in the study, both Cox Regression and Kaplan–Meier analyses were selected. Cox regression analysis was selected for the initial univariate evaluation since this enables studying and presenting large data quantities and further multivariate analysis. In a multivariate setting, Cox proportional hazard models were adjusted for sex, tumor histology (adenocarcinoma or other), and the selected first‐line EGFR TKI (gefitinib/afatinib vs. erlotinib). The confidence level of 95% was considered significant. Kaplan–Meier analyses with log‐rank tests were used to compare survival differences. Pearson's Chi‐square test was used to determine association between groups. All statistical analyses were performed using IBM SPSS Statistics V.29.0 for Windows in a secure portal platform (Kapseli) provided by Findata.

## 3 Results

### 3.1 Population Demographics

Demographics of the patients are presented in Table 1. Since the study was based on national registries with scarce clinical information, baseline demographics are limited. Therefore, all possible confounding factors cannot be controlled.

**TABLE 1:** Patient demographics.

	*N* (%)
All	1494 (100)
Sex	
Female	784 (52.5)
Male	710 (47.5)
Stage	
Local	68 (4.6)
Advanced	1018 (68.1)
Unknown	408 (27.3)
Histology	
Adenocarcinoma	1101 (73.7)
Other	393 (26.3)
First EGFR TKI	
Gefitinib	238 (15.9)
Erlotinib	998 (66.8)
Afatinib	258 (17.3)

Analgesic purchases^a^	−14 days to +14 days		−14 days to +180 days	
	*n*	%	*n*	%
NSAID	90	6.0	432	28.9
Acetaminophen	171	11.4	592	39.6
Weak opioids	112	7.5	438	29.3
Strong opioids	332	22.2	484	32.4
Immunomodulatory	66	4.4	95	6.4
Nonimmunomodulatory	299	20.0	460	30.8

Abbreviations: EGFR TKI, epidermal growth factor receptor tyrosine kinase inhibitor; NSAID, nonsteroidal anti‐inflammatory drugs.

^a^ 0 day being the 1st EGFR TKI purchase date.

Of the study population (*n* = 1494), 52.5% were female. Most patients (*n* = 1018, 68.1%) had advanced stage at diagnosis and adenocarcinoma histology (*n* = 1101, 73.7%). Erlotinib was the most prescribed EGFR TKI (*n* = 998, 66.6%), followed by afatinib (*n* = 258, 17.2%) and gefitinib (*n* = 238, 15.9%). During the study period (2011–2020), osimertinib was not a first‐line standard, and these patients were excluded from the final analysis (*n* = 4) (Table 1).

At the initiation of EGFR TKI treatment (−14 days to +14 days from the first TKI purchase), 6% (*n* = 90) of patients had NSAID, 11.4% (*n* = 171) acetaminophen, 7.5% (*n* = 112) weak opioid, and 22.2% (*n* = 332) strong opioid purchases. Purchases of analgesics were more frequent throughout the whole study period (−14 days to +180 days from the first TKI purchase), NSAIDs in 28.9% (*n* = 432), acetaminophen in 39.6% (*n* = 592), weak opioids in 29.3% (*n* = 438), and strong opioids in 32.4% (*n* = 484) of the study population (Table 1). Concurrent use of nonopioid analgesics with strong opioids was common; 36.6% (*n* = 33) and 43.2% (*n* = 74) of NSAID and acetaminophen users had concurrent strong opioid purchases at the initiation period (−14 to +14 days). Concomitant use of weak and strong opioids (*n* = 18) was less frequent (not shown).

In the generated EGFR mutant cohort (*n* = 466), the use of analgesics was less common than in the whole cohort (Table S1). Due to the small sample size, no further survival analysis was carried out in this cohort.

### 3.2 Time‐on‐Treatment and Overall Survival According to the Use of Analgesics

Next, we studied the association of analgesics to the TKI ToT and OS. In the univariate analysis for ToT, NSAIDs were not associated with ToT while inferior ToT was observed with concurrent purchases (−14 days to +14 days from the first TKI purchase) of acetaminophen (HR 1.242, 95% CI 1.053–1.48), weak opioids (HR 1.397, 95% CI 1.144–1.706), and strong opioids (HR 1.467, 95% CI 1.293–1.665). In the multivariate analysis for ToT including sex, histology, and the first TKI, only opioids retained their association with an emphasis on strong opioids (HR 1.454, 95% CI 1.276–1.656) compared to the weak opioids (HR 1.368, 95% CI 1.119–1.674) (Table 2).

**TABLE 2:** Univariate and multivariate analyses for time‐on‐treatment and survival in the whole cohort according to the use of analgesics.

	Univariate		Multivariate	
	HR	95% CI	HR	95% CI
**Time‐on‐treatment**				
Sex				
Female vs. male	0.748	0.673–0.831	0.813	0.731–0.906
Histology				
Adenocarcinoma vs. other	0.676	0.600–0.761	0.764	0.676–0.863
First EGFR TKI				
Gefitinib/afatinib vs. erlotinib	0.544	0.485–0.612	0.585	0.519–0.659
NSAID				
Yes vs. no	0.919	0.817–1.033		
−14 days to +14 days yes vs. no	1.191	0.955–1.485		
Acetaminophen				
Yes vs. no	1.061	0.953–1.182		
−14 days to +14 days yes vs. no	1.242	1.053–1.484	1.152	0.972–1.366
Weak opioids				
Yes vs. no	1.173	1.045–1.316		
−14 days to +14 days yes vs. no	1.397	1.144–1.706	1.368	1.119–1.674
Strong opioids				
Yes vs. no	1.496	1.336–1.675		
−14 days to +14 days yes vs. no	1.467	1.293–1.665	1.454	1.276–1.656
**Survival**				
Sex				
Female vs. male	0.731	0.654–0.817	0.787	0.703–0.882
Histology				
Adenocarcinoma vs. other	0.665	0.587–0.754	0.750	0.660–0.852
First EGFR TKI				
Gefitinib/afatinib vs. erlotinib	0.543	0.478–0.612	0.584	0.512–0.665
NSAID				
Yes vs. no	0.992	0.876–1.123		
−14 days to +14 days yes vs. no	1.322	1.051–1.664	1.208	0.956–1.526
Acetaminophen				
Yes vs. no	1.164	1.038–1.305		
−14 days to +14 days yes vs. no	1.420	1.197–1.685	1.261	1.056–1.506
Weak opioids				
Yes vs. no	1.257	1.113–1.420		
−14 days to +14 days yes vs. no	1.420	1.153–1.748	1.290	1.043–1.595
Strong opioids				
Yes vs. no	1.624	1.442–1.830		
−14 days to +14 days yes vs. no	1.735	1.518–1.983	1.690	1.471–1.940

Abbreviations: EGFR TKI, epidermal growth factor receptor tyrosine kinase inhibitor; NSAID, nonsteroidal anti‐inflammatory drugs.

In the univariate for OS, all the analgesic purchases excluding NSAIDs were associated with inferior survival during the entire study period (−14 days to +180 days). In the multivariate analysis for OS, acetaminophen (HR 1.261, 95% CI 1.056–1.506), weak opioids (HR 1.290, 95% CI 1.043–1.595), and most notably strong opioids (HR 1.690, 95% CI 1.471–1.940) retained their association with inferior survival (Table 2).

### 3.3 Time‐on‐Treatment and Overall Survival According to the Use of Immunomodulatory and Nonimmunomodulatory Opioids

Since strong opioids have variable immunomodulatory effects which might affect outcomes, we carried out a ToT and survival analysis according to this grouping: immunomodulatory (morphine, fentanyl) and nonimmunomodulatory (oxycodone, hydromorphone). Purchases of nonimmunomodulatory opioids were found in 20% (*n* = 299) in the time period of −14 days to +14 days and 30.8% (*n* = 460) in −14 days to +180 days, while immunomodulatory opioid use was less common, 4.4% (*n* = 66) and 6.4% (*n* = 95), respectively (Table 1).

In the univariate analysis, immunomodulatory opioids showed inferior ToT compared to nonimmunomodulatory (HR 1.458, 95% CI 1.158–1.836) in the whole study period (−14 days to +180 days) but not in the initiation period (−14 days to +14 days), possibly due to the small number (*n* = 66) of immunomodulatory opioid purchasers in the beginning of the EGFR TKI treatment. The statistical significance of ToT stratified by the immunomodulatory class of opioid remained in multivariate analysis (HR 1.448, 95% CI 1.148–1.826) including sex, histology, and the first TKI. In the univariate analysis for OS, immunomodulatory opioids showed similar unfavorable outcomes (HR 1.509, 95% CI 1.184–1.924, −14 days to +180 days) which was also observed in the multivariate analysis (HR 1.479, 95% CI 1.158–1.888) (Table 3).

### 3.4 Outcomes According to Concurrent Use of Nonopioids and Strong Opioids

**FIGURE 1:**
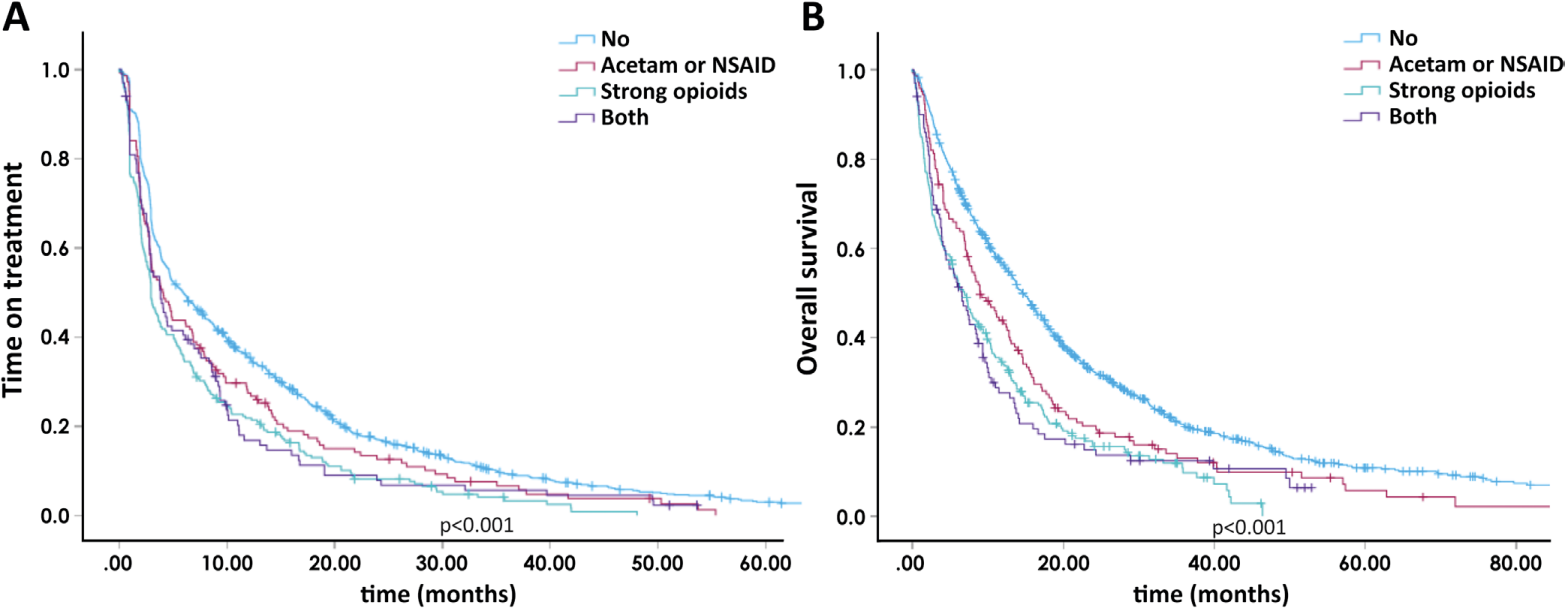
Time‐on‐treatment (A) and overall survival (B) according to the use of analgesics grouping.

Since strong opioids were found to heavily impact ToT and OS, we further investigated the effect of concurrent analgesics use according to a grouping of nonopioids, strong opioids, both, and no analgesics purchases. In ToT analysis with Kaplan–Meier, there was a significant difference (*p* < 0.001) according to our analgesics grouping (Figure 1A). In the univariate Cox regression analysis, a nonsignificant trend for inferior survival in strong opioid (HR 1.234, 95% CI 0.995–1.530) and strong opioid + NSAID/acetaminophen users was observed (HR 1.115, 95% CI 0.856–1.453) compared to nonopioid users (not shown). In the survival analysis, we also detected a significant difference (*p* < 0.001) according to our analgesics grouping (Figure 1B). In the univariate Cox regression analysis, a significant survival difference in strong opioid users (HR 1.283, 95% CI 1.024–1.609) was seen compared to nonopioid users. Among the strong opioid + NSAID/acetaminophen users, a similar trend was observed, but this was statistically nonsignificant (HR 1.281, 95% CI 0.972–1.689) (not shown).

### 3.5 Multivariate Analysis With Strong Opioids and Other Drugs Known to Effect ToT and OS Outcomes

We have previously reported a positive association to ToT and OS with tetracycline antibiotics and topical corticosteroids [5, 6]. In addition, existing data suggest that there is a negative association to treatment outcomes with drugs for acid‐related disorders among the EGFR TKI users [4]. To further assess the association of strong opioids to ToT and OS, we carried out a univariate and multivariate analyses according to the use of supportive medications. In a multivariate analysis for ToT including sex, histology, and first TKI, purchases of tetracyclines, topical corticosteroids, and drugs for acid‐related disorders, significance was retained for the strong opioid users (HR 1.479, 95% CI 1.301–1.682). In the multivariate analysis for OS with the same variables included, strong opioids retained their association to inferior survival (HR 1.752, 95% CI 1.530–2.006). Interestingly, drugs for acid‐related disorders lost their significant association to survival in this multivariate analysis (HR 1.096, 95% CI 0.926–1.296). We hypothesized that there might be an association between the use of strong opioids and drugs for acid‐related disorders. As expected, we observed an association between purchases of strong opioids and drugs for acid‐related disorders (*p* < 0.001), while no association was detected for strong opioids and tetracyclines or topical corticosteroids (not shown) (Table 4).

**TABLE 3:** Univariate and multivariate analyses for time‐on‐treatment and survival according to the use of strong opioids either in immunomodulatory or nonimmunomodulatory categories.

	Univariate		Multivariate	
	HR	95% CI	HR	95% CI
**Time‐on‐treatment**				
Sex				
Female vs. male	0.748	0.673–0.831	0.689	0.572–0.830
Histology				
Adenocarcinoma vs. other	0.676	0.600–0.761	0.641	0.518–0.795
First EGFR TKI				
Gefitinib/afatinib vs. erlotinib	0.544	0.485–0.612	0.604	0.489–0.745
Strong opioid class				
Immunomodulatory vs. nonimmunomodulatory	1.458	1.158–1.836	1.448	1.148–1.826
−14 days to +14 days immunomodulatory vs. nonimmunomodulatory	1.350	0.969–1.881		
**Survival**				
Sex				
Female vs. male	0.731	0.654–0.817	0.713	0.587–0.867
Histology				
Adenocarcinoma vs. other	0.665	0.587–0.754	0.672	0.539–0.837
First EGFR TKI				
Gefitinib/afatinib vs. erlotinib	0.543	0.478–0.612	0.573	0.457–0.720
Strong opioid class				
Immunomodulatory vs. nonimmunomodulatory	1.509	1.184–1.924	1.479	1.158–1.888
−14 days to +14 days immunomodulatory vs. nonimmunomodulatory	1.133	0.800–1.605		

Abbreviations: EGFR TKI, epidermal growth factor receptor tyrosine kinase inhibitor; NSAID, nonsteroidal anti‐inflammatory drugs.

**TABLE 4:** Univariate and multivariate analyses for time‐on‐treatment and survival according to the use tetracyclines, topical corticoids, drugs for acid‐related disorders and strong opioids.

	Univariate		Multivariate	
	HR	95% CI	HR	95% CI
**Time‐on‐treatment**				
Sex				
Female vs. male	0.748	0.673–0.831	0.820	0.737–0.913
Histology				
Adenocarcinoma vs. other	0.676	0.600–0.761	0.765	0.677–0.865
First EGFR TKI				
Gefitinib/afatinib vs. erlotinib	0.544	0.485–0.612	0.575	0.511–0.648
Tetracyclines				
−14 days to +14 days yes vs. no	0.857	0.750–0.979	0.883	0.771–1.011
Corticosteroids				
−14 days to +14 days yes vs. no	0.803	0.674–0.958	0.752	0.628–0.901
Drugs for acid‐related disorders				
−14 days to +14 days yes vs. no	1.271	1.086–1.488	1.199	1.022–1.405
Strong opioids				
−14 days to +14 days yes vs. no	1.467	1.293–1.665	1.479	1.301–1.682
**Survival**				
Sex				
Female vs. male	0.748	0.673–0.831	0.794	0.709–0.888
Histology				
Adenocarcinoma vs. other	0.676	0.600–0.761	0.768	0.676–0.872
First EGFR TKI				
Gefitinib/afatinib vs. erlotinib	0.544	0.485–0.612	0.575	0.504–0.655
Tetracyclines				
−14 days to +14 days yes vs. no	0.743	0.642–0.860	0.729	0.630–0.845
Corticosteroids				
−14 days to +14 days yes vs. no	0.869	0.722–1.046		
Drugs for acid‐related disorders				
−14 days to +14 days yes vs. no	1.210	1.025–1.428	1.096	0.926–1.296
Strong opioids				
−14 days to +14 days yes vs. no	1.735	1.518–1.983	1.752	1.530–2.006

Abbreviation: EGFR TKI, epidermal growth factor receptor tyrosine kinase inhibitor.

## 4 Discussion

To our knowledge, this study is the first one to examine the clinical outcomes of NSCLC patients treated concurrently with EGFR TKIs and different analgesics. Our results reveal that opioid use was associated with worse outcomes, which is consistent with other studies worldwide [8, 17, 18, 19, 20]. In our study, however, we found these results to be more pronounced according to the immunomodulatory class and the higher strength of the opioid. In general, strong opioids had a paramount effect on ToT and OS, while the outcomes of concurrent weak opioid users resembled more those of nonopioids.

Contradictory to previous studies, we found no association between treatment outcomes and NSAID use in advanced NSCLC treated with EGFR TKIs [13, 14]. This discrepancy could be explained by different study populations or concurrent pharmacodynamics of EGFR TKI and NSAIDs. NSAIDs inhibit COX enzymes, which result in a decreased amount of prostaglandin E2 (PGE2), a molecule associated with protumorigenic properties. Theoretically, when NSAIDs are used in conjunction with other antitumor drugs, decreasing PGE2 levels could provide additive means to impair tumor growth [11, 12]. This is not inevitably true when NSAIDs are used concurrently with EGFR TKIs: PGE2 induces EGFR activation and nuclearization through EGFR tyrosine kinase [24]. Essentially, EGFR TKIs could already inflict the effects NSAIDs might have on the tumor cell, which would explain the lack of survival benefits found in our study. Additionally, EGFR‐mutated patients with high serum COX‐2 expression have been shown to have higher response rates and improved PFS [25]. In our cohort, the number of patients in the artificial EGFR mutant cohort who had purchased analgesics was low, and therefore, no reliable outcome analysis could be carried out in the sub‐cohort.

Interestingly, opioids have variable immune effects depending on their class, and this effect was also investigated in the current study [21, 22, 23]. The main binding site of an opioid is the μ‐opioid receptor (MOR). MOR has been associated with several protumorigenic properties: the co‐activation of EGFR, the promoting of epithelial mesenchymal transition (EMT) which gives tumor cells their migrative and invasive properties, and the activation of various other tumor‐promoting pathways outside of EGFR [20, 26, 27, 28, 29, 30, 31]. For immune cells, the activation of MOR induced an immunosuppressive state by reducing natural killer (NK) cell activity and decreasing lymphocyte proliferation, which was reversible by MOR antagonist [21]. While all opioids use MOR as a binding site, their effects on immunity vary. The exact reason behind this is still unknown, but there are hypotheses involving the differences in opioids' chemical structures, which affect their MOR affinity and selectiveness [20, 21, 22, 23]. At a normal antinociceptive dose, morphine and fentanyl have a high affinity to MOR and also suppress NK cells, lymphocyte proliferation, and cytokine production. In contrast, oxycodone and hydromorphone do not share this effect [21, 22, 23].

In our study, we found that immunomodulatory opioids are associated with poor ToT and OS compared to nonimmunomodulatory. Even though EGFR–altered NSCLC is generally not considered as an immunologically “hot” tumor subtype due to low responses to anti‐PD‐(L)1 therapies, cancer reaches past T cells [32]. Furthermore, our cohort included mainly EGFR wild‐type patients treated with later line erlotinib and is not reflective of EGFR mutant disease. International guidelines generally recommend morphine as the first‐line treatment of moderate or severe pain [7, 33]. Contradictorily, our results favor using oxycodone or other nonimmunomodulatory opioid analgesics, since they might have a more favorable immune profile [7, 20, 21, 22].

Pain and opioid usage have both been independently associated with worse survival in NSCLC. Patients with low pain and low daily dose of opioids had superior survival compared to those with high doses of opioids or high pain [8]. Pain is known to induce systemic immunosuppression through the hypothalamic–pituitary–adrenal axis, but whether pain itself is tumorigenic or is just an indicator of a worse cancer situation remains undecided [21]. This also raises the question: Is pain itself more harmful than the analgesic? More in‐depth research is needed to assess the clinical association with analgesics and tumor progression to avoid possible treatment pitfalls.

Cancer patients requiring opioids are usually more symptomatic and thus could reflect a subgroup with more advanced disease, worse PS, and poor prognosis. We present here a hypothesis that the use of strong opioids could serve as an artificial PS indicator in situations where no PS data is otherwise available, a common scenario when working with registry data not extracted directly from medical records. Further studies with available PS data are required to validate this hypothesis.

Our study has its obvious limitations generated mainly by the retrospective nature. Due to the register limitations, the study lacks data on important variables such as EGFR mutation status, PS, or line of systemic therapy. This brings forth the discussion of whether our findings are simply explained by the selection bias. While we have no absolute answers, our real‐world findings align with hypotheses derived from preclinical studies and showcase the paucity of research around this subject. Additionally, due to register limitations, the use of opioid classes (e.g., immunomodulatory vs. nonimmunomodulatory) and patient subgroups (e.g., artificial EGFR mutant cohort) was limited in number, which prevented us from making strong conclusions. Since our cohort was based on reimbursed purchases, we cannot certainly conclude that all purchased medications are used. Oncological patients usually have a very high adherence to prescribed medications, and we therefore believe that purchase is a precise indicator of drug use. However, in Finland, there is a significant use of over‐the‐counter nonopioid analgesics, which generates uncertainties in these medication classes.

## 5 Conclusions

In EGFR TKI‐treated NSCLC, opioids are an independent risk factor for worse ToT and OS. The outcomes differed by the immunomodulatory category of opioids, suggesting that analgesics class can potentially have a direct impact on EGFR TKI effects. Contrary to previous results in other lung cancer cohorts, NSAIDs did not provide outcome benefits.

## Author Contributions

**Laura S. Puuniemi:** conceptualization (equal), formal analysis (supporting), funding acquisition (equal), visualization (supporting), writing – original draft (lead), writing – review and editing (lead). **Sanna M. E. Iivanainen:** conceptualization (equal), investigation (equal), validation (equal), writing – original draft (supporting), writing – review and editing (equal). **Martti Arffman:** data curation (lead), writing – review and editing (equal). **Riitta L. Kaarteenaho:** project administration (supporting), supervision (supporting), validation (equal), writing – review and editing (equal). **Jussi P. Koivunen:** conceptualization (lead), formal analysis (lead), funding acquisition (equal), investigation (equal), project administration (lead), resources (lead), supervision (lead), validation (equal), visualization (lead), writing – original draft (equal), writing – review and editing (equal).

## Ethics Statement

All data collection was carried out according to national legislation and under a permit from the Findata (THL/6637/14.05.22/2021). Pseudonymization was carried out before data analysis.

## Consent

The authors have nothing to report.

## Conflicts of Interest

L.S.P. declares no conflicts of interest. M.A. reports research support from Roche outside the submitted work. S.M.E.I. reports personal fees from MSD, personal fees and institutional grants from Roche, personal fees from BMS, personal fees and institutional grants from AstraZeneca, personal fees from Novartis, personal fees from Takeda, personal fees from Janssen, personal fees from Eisai all outside the submitted work. R.L.K. reports consulting, lecture, and advisory board fees from Boehringer Ingelheim; a virtual congress cost from Novartis; an advisory board fee from MSD, outside the submitted work. J.P.K. reports personal fees from Roche, personal fees from AstraZeneca, personal fees from Janssen, personal fees from BMS, personal fees from Merck, personal fees from Amgen, personal fees from Novartis, personal fees from Merck KgA, lecturing fees from Siemens Heatlhineers, personal fees from Sanofi, personal fees from Janssen, and Pfizer all outside the submitted work. J.P.K. is a former part‐time employee at Faron Pharmaceuticals.

## Supporting information


**Table S1.** Analgesics purchases (−14 to +14 days) in artificial EGFR mutant cohort.

## Data Availability

Owing to data protection legislation in Finland, individual‐level data on the study subjects cannot be released.
