# Supplementary material for: Concomitant Use of Analgesics and EGFR TKIs in Lung Cancer Patients: Outcomes and Perspectives From a Finnish Retrospective Register–Based Study
Source: Cancer Med. 2025 Jul 12;14(13):e71040. doi: 10.1002/cam4.71040 (PMC12254695; doi:10.1002/cam4.71040)
Supplement: Supplementary file 1 — Table S1. Analgesics purchases (−14 to +14 days) in artificial EGFR mutant cohort. [file CAM4-14-e71040-s001.docx]

**Supplementary table 1**. Analgesics purchases (-14 to + 14d) in artificial EGFR mutant cohort

| Analgesic purchases^a^ | -14d to +14d | |
| --- | --- | --- |
|  | n | % |
| NSAID | 12 | 2.6 |
| Acetaminophen | 27 | 5.8 |
| Weak opioids | 28 | 6.0 |
| Strong opioids | 90 | 19.3 |
| Immunomodulatory | 12 | 2.6 |
| Non-immunomodulatory | 78 | 16.7 |

*Individuals categorized by their highest analgesic purchases according to WHO pain ladder

Abbreviations: NSAID, non-steroidal anti-inflammatory drugs
